# Cardiovascular Alterations in Primary Hyperparathyroidism: Associations with Risk Profile, Cardiac Structure, and Function

**DOI:** 10.3390/jcm15176605

**Published:** 2026-08-26

**Authors:** Desislava Zamfirova, Mihail Boyanov, Stefan Naydenov

**Affiliations:** 1 Endocrinology Clinic, UMHAT “Alexandrovska”, Medical University of Sofia, 1431 Sofia, Bulgaria; dzamfirova@gmail.com (D.Z.); mihailboyanov@yahoo.com (M.B.); 2 Department of Internal Diseases “Prof. St. Kirkovich”, Medical University of Sofia, 1431 Sofia, Bulgaria

**Keywords:** primary hyperparathyroidism, cardiovascular risk, left ventricular hypertrophy, pulmonary artery systolic pressure

## Abstract

**Background/Objectives**: Primary hyperparathyroidism (PHPT) has been associated with cardiovascular abnormalities, but the extent to which these reflect PHPT or conventional risk factors remains uncertain. We compared cardiovascular risk profiles and cardiac findings in patients with PHPT and controls. **Methods**: In this single-center cross-sectional case–control study, 5868 hospitalized patients were screened between January 2024 and December 2025. Fifty-four patients with biochemically confirmed PHPT and 23 age- and sex-comparable controls underwent standardized clinical, laboratory, 12-lead electrocardiogram (ECG), 24-h Holter ECG, and transthoracic echocardiographic assessment. **Results**: Patients with PHPT had higher systolic blood pressure (132.5 vs. 130.0 mmHg; *p* = 0.049), more type 2 diabetes (18.5% vs. 0%; *p* = 0.028), left ventricular hypertrophy (57.4% vs. 30.4%; *p* = 0.046), longer transmitral flow deceleration time (281.5 vs. 222.0 ms; *p* < 0.001), higher pulmonary artery systolic pressure (PASP) (24.5 vs. 19.0 mmHg; *p* < 0.001), and more aortic valve sclerosis/fibrotic thickening (38.9% vs. 13.0%; *p* = 0.032). High/very high cardiovascular risk was present in 54/54 (100%) PHPT patients versus 20/23 (86.96%) controls (*p* = 0.024). After adjustment, PHPT remained associated with longer deceleration time, higher PASP, aortic valve sclerosis/fibrotic thickening, and pericardial effusion. **Conclusions**: PHPT was associated with selected echocardiographic abnormalities, but causality cannot be inferred. Larger prospective studies are needed.

## 1. Introduction

Primary hyperparathyroidism (PHPT) is a common endocrine disorder characterized by autonomous secretion of parathyroid hormone (PTH), resulting in hypercalcemia and disturbances in calcium–phosphate metabolism [1,2]. It is the third most common endocrine disease after type 2 diabetes mellitus (T2DM) and thyroid disorders. The estimated prevalence ranges from 0.1% to 1% in the general population and increases substantially with age, particularly among postmenopausal women [1,3].

Although PHPT has traditionally been viewed as a disorder affecting the skeletal and renal systems, increasing evidence indicates that it also has important cardiovascular consequences [4,5,6]. Elevated circulating PTH levels and chronic exposure to hypercalcemia may adversely affect the cardiovascular system through several pathophysiological mechanisms [3,5,7]. Chronic hypercalcemia may contribute to cardiovascular alterations through several complementary mechanisms. Sustained increases in extracellular calcium can modify vascular smooth-muscle tone and intracellular calcium handling, potentially promoting vasoconstriction and increased vascular stiffness [6,8,9,10]. Hypercalcemia may also favor endothelial dysfunction and, particularly when more pronounced or prolonged, facilitate calcium deposition and osteogenic remodeling within vascular and valvular tissues [6,8,9,10]. At the myocardial level, altered calcium homeostasis may affect excitation–contraction coupling and electrical conduction, thereby potentially influencing myocardial relaxation and arrhythmogenesis [9,10,11]. These calcium-mediated effects may coexist and interact with the direct cardiovascular actions of excess PTH, making their individual contributions difficult to distinguish in PHPT [4,8,11,12].

Experimental and clinical studies suggest that excessive PTH secretion promotes myocardial hypertrophy, interstitial fibrosis, endothelial dysfunction, vascular stiffness, oxidative stress, and chronic low-grade inflammation [4,13,14]. In addition, PTH may activate both the renin–angiotensin–aldosterone system and the sympathetic nervous system [9,12,15]. The presence of PTH receptors in cardiomyocytes and vascular smooth muscle cells further supports a direct effect of PTH on cardiovascular tissues [9,16].

A growing body of evidence has linked PHPT to an increased prevalence of cardiovascular disease (CVD) [4,8,17]. Patients with PHPT have been reported to exhibit higher rates of arterial hypertension (HTN), left ventricular (LV) hypertrophy, impaired diastolic function, coronary artery disease, heart failure (HF), atrial fibrillation (AF), and cerebrovascular disease [18,19,20]. Furthermore, PHPT has been associated with increased cardiovascular mortality [9,21]. This association has been supported by large observational cohorts and, more recently, by a systematic review and meta-analysis including more than 260,000 patients with PHPT, in which PHPT was associated with a 61% higher risk of cardiovascular death and a 39% higher risk of all-cause mortality compared with the general population [11]. Although the available studies are heterogeneous and predominantly observational, these findings suggest that the cardiovascular implications of PHPT may extend beyond subclinical structural and functional abnormalities to clinically relevant long-term outcomes. Notably, elevated PTH concentrations have been associated with adverse cardiovascular outcomes even in the absence of overt hypercalcemia [10,21,22]. This observation suggests that PTH itself may contribute to cardiovascular risk independently of serum calcium levels.

Despite accumulating evidence, the cardiovascular consequences of PHPT remain incompletely understood. Available studies have reported inconsistent findings regarding the extent and clinical significance of cardiovascular involvement, particularly in patients with mild or asymptomatic disease [5,6,8]. Furthermore, comprehensive evaluations integrating cardiovascular risk factors, established cardiovascular disease, electrocardiographic abnormalities, and detailed echocardiographic findings remain limited. As a result, it is still unclear whether the increased cardiovascular involvement associated with PHPT is entirely attributable to conventional risk factors or reflects disease-specific effects of chronic PTH excess [5,21,22].

Another area of ongoing debate is the reversibility of cardiovascular abnormalities following parathyroidectomy. While several studies have demonstrated improvements in blood pressure (BP), cardiac structure, vascular function, and cardiovascular risk markers after surgery, others have reported only modest or inconsistent benefits [2,4,13,20]. Therefore, the extent to which cardiovascular alterations associated with PHPT are reversible remains uncertain [5,23,24].

Given these unresolved issues, further characterization of the cardiovascular profile associated with PHPT is warranted. The aim of the present study was to compare cardiovascular risk profiles, comorbidities, laboratory, electrocardiogram (ECG) and echocardiographic characteristics between patients with PHPT and controls, and to identify clinical, biochemical, ECG, and echocardiographic factors associated with cardiovascular abnormalities in this endocrine disorder.

## 2. Materials and Methods

### 2.1. Study Design and Population

This single-center cross-sectional case–control study was conducted between 1 January 2024 and 31 December 2025. During the study period, all consecutive patients hospitalized at the Endocrinology Clinic (*n* = 5868) were prospectively screened for eligibility. Patient selection was not based on cardiovascular symptoms, cardiovascular comorbidities, or any predefined pattern of cardiovascular abnormalities. Patients were considered eligible for the PHPT group when biochemical evaluation confirmed PHPT according to the predefined diagnostic criteria and all study inclusion and exclusion criteria were fulfilled. Of the 5868 screened patients, 54 patients (0.9%) with confirmed PHPT met these criteria and were included in the final study cohort.

A control group comprising 23 individuals without biochemical or clinical evidence of PHPT was recruited during the same study period. Controls were selected to be comparable with the PHPT group in terms of age and sex and underwent the same standardized cardiovascular assessment. Control recruitment was based on the predefined study protocol and the availability of eligible individuals fulfilling the control-group criteria; therefore, a fixed 1:1 matching ratio was not applied. No additional controls were selected retrospectively after completion of enrollment in order to avoid post hoc selection and the potential introduction of selection bias. Matching was not performed for other cardiovascular risk factors or comorbidities. Consequently, between-group differences in variables potentially related to cardiac remodeling, including systolic blood pressure and T2DM, were considered potential confounders and were accounted for in the multivariable analyses where appropriate.

The diagnosis of PHPT was established according to the recommendations of the Fifth International Workshop on the Evaluation and Management of Primary Hyperparathyroidism and the current European Expert Consensus on Parathyroid Disorders [2,25]. The median age was 61 years (IQR 53–70 years) in the PHPT group and 60 years (IQR 55–69 years) in the control group, with no significant difference between groups (*p* = 0.969). Women predominated in both groups, accounting for 88.9% of patients with PHPT and 95.7% of controls, respectively, with a comparable sex distribution (*p* = 0.667).

### 2.2. Inclusion and Exclusion Criteria

Identification of eligible PHPT patients was based exclusively on predefined biochemical diagnostic criteria and was independent of the presence or absence of cardiovascular abnormalities. Participants were eligible for inclusion in the PHPT group if they: (1) were ≥18 years of age; and (2) had biochemically confirmed PHPT diagnosed according to the recommendations of the Fifth International Workshop on the Evaluation and Management of Primary Hyperparathyroidism and the European Expert Consensus on Parathyroid Disorders [2,25].

Control subjects were eligible if they were ≥18 years of age and had no clinical or biochemical evidence of PHPT, including normal serum calcium and parathyroid hormone concentrations.

Participants were excluded from both groups if they had (1) secondary or tertiary hyperparathyroidism; (2) an acute cardiovascular event within the preceding three months, including myocardial infarction, stroke, or acute HF; (3) active malignant disease; (4) severe systemic inflammatory or infectious disorders; or (5) incomplete clinical, laboratory, ECG, or echocardiographic data required for the analysis.

All clinical, laboratory, ECG, and echocardiographic assessments were performed during a single study visit. Given the cross-sectional study design, no longitudinal follow-up was performed.

### 2.3. Clinical Assessment

All participants underwent a standardized clinical evaluation at study inclusion. Demographic characteristics, anthropometric measurements, cardiovascular risk factors, medical history, comorbidities, and current pharmacological therapy were systematically recorded.

Body mass index (BMI) was calculated as weight in kilograms divided by height in meters squared (kg/m^2^). Office BP was measured three consecutive times after at least 5 min of rest in the sitting position using a validated automated sphygmomanometer. Measurements were initially obtained in both arms, and the average of the last two measurements from the arm with the higher BP was used for analysis.

Information regarding cardiovascular risk factors, including HTN, T2DM, dyslipidemia, smoking status, and obesity, as well as previous CVD, including coronary artery disease, HF, cerebrovascular disease, and AF, was obtained from medical records and structured patient interviews.

### 2.4. Electrocardiographic Assessment

A standard 12-lead ECG was recorded in all participants at study inclusion using a Mortara ELI 280c electrocardiograph (Mortara Instrument Inc., Milwaukee, WI, USA). ECG recordings were analyzed for heart rhythm, heart rate, conduction abnormalities, evidence of previous myocardial infarction, and ECG signs of cardiac chamber enlargement in accordance with current international guidelines.

Twenty-four-hour Holter ECG monitoring was performed in all participants using a 7-channel digital Holter recorder (Medilog Darwin V2, Schiller Ltd., Baar, Switzerland). Holter ECG recordings were analyzed to assess heart rhythm, heart rate, supraventricular and ventricular arrhythmias, and conduction disturbances according to current international recommendations.

The ECG parameters were automatically measured from the Holter ECG recordings using dedicated software for ECG analysis.

### 2.5. Echocardiographic Assessment

All participants underwent transthoracic echocardiography performed by an experienced cardiologist using standard imaging protocols. Examinations were performed using a General Electric Vivid E95ie (GE HealthCare Technologies, Inc., Chicago, IL, USA) ultrasound system equipped with a 4Vc matrix-array sector transducer (GE HealthCare Technologies, Inc., Chicago, IL, USA) and were conducted in accordance with the recommendations of the European Association of Cardiovascular Imaging [26].

Cardiac chamber dimensions, LV geometry, systolic function, and diastolic function were assessed using standard two-dimensional, M-mode, and Doppler techniques.

Measurements included left atrial (LA) and right atrial (RA) diameter, LV end-diastolic and end-systolic dimensions and volumes, right ventricular diameter (RV), interventricular septum (IVS) thickness and left ventricular posterior wall (LVPW) thickness, and pulmonary artery systolic pressure (PASP). Left ventricular ejection fraction (LVEF) was determined using the biplane Simpson method.

Diastolic function was assessed using transmitral Doppler flow velocities and tissue Doppler imaging parameters. LV diastolic dysfunction was graded according to standard echocardiographic criteria. Grade I diastolic dysfunction was defined as impaired LV relaxation with normal or low filling pressures; Grade II as a pseudonormal filling pattern associated with elevated LV filling pressures; and Grade III as a restrictive filling pattern characterized by markedly impaired ventricular compliance and elevated filling pressures. Classification was based on an integrated assessment of transmitral E- and A-wave velocities and E/A ratio, mitral annular e′ velocity, E/e′ ratio, left atrial size, and other supportive Doppler parameters, as appropriate [27,28].

In addition, global longitudinal strain (GLS) and other advanced echocardiographic parameters were evaluated in all participants.

Aortic and mitral valve structural abnormalities were assessed qualitatively by transthoracic echocardiography. Valve sclerosis/fibrotic thickening was defined by increased leaflet thickness and echogenicity; valvular calcification was not separately quantified or graded using a dedicated calcification score.

Pericardial effusion was assessed qualitatively by transthoracic echocardiography and recorded as present or absent.

### 2.6. Laboratory Investigations

Venous blood samples were obtained during routine clinical evaluation. Laboratory analyses included serum calcium, phosphate, PTH, creatinine, glucose, lipid profile, and other routinely assessed biochemical parameters.

### 2.7. Cardiovascular Risk Assessment

Cardiovascular risk was assessed in accordance with the 2021 European Society of Cardiology (ESC) Guidelines on cardiovascular disease prevention in clinical practice [29].

For participants younger than 70 years without established atherosclerotic cardiovascular disease (ASCVD), T2DM, chronic kidney disease (CKD), familial hypercholesterolaemia, or other conditions conferring high or very high cardiovascular risk, the 10-year risk of fatal and non-fatal cardiovascular events was estimated using the SCORE2 algorithm. SCORE2 incorporates age, sex, smoking status, systolic BP, and non-high-density lipoprotein cholesterol and is calibrated according to the cardiovascular risk of the country of residence.

Participants with established ASCVD, T2DM, CKD, familial hypercholesterolaemia, or other high-risk conditions were classified into the appropriate cardiovascular risk category according to the 2021 ESC Guidelines rather than by SCORE2 estimation.

### 2.8. Ethical Considerations

The study was conducted in accordance with the ethical principles of the Declaration of Helsinki and Good Clinical Practice guidelines. Written informed consent was obtained from all participants before enrollment. The study protocol was registered in the ClinicalTrials.gov Protocol Registration and Results System under the protocol identifier PHPT-CVD-2023. The requirement for ethical approval was waived because of the cross-sectional, non-interventional design of the study and the use of fully anonymized clinical data, in accordance with applicable national regulations and institutional requirements.

**Figure 1:**
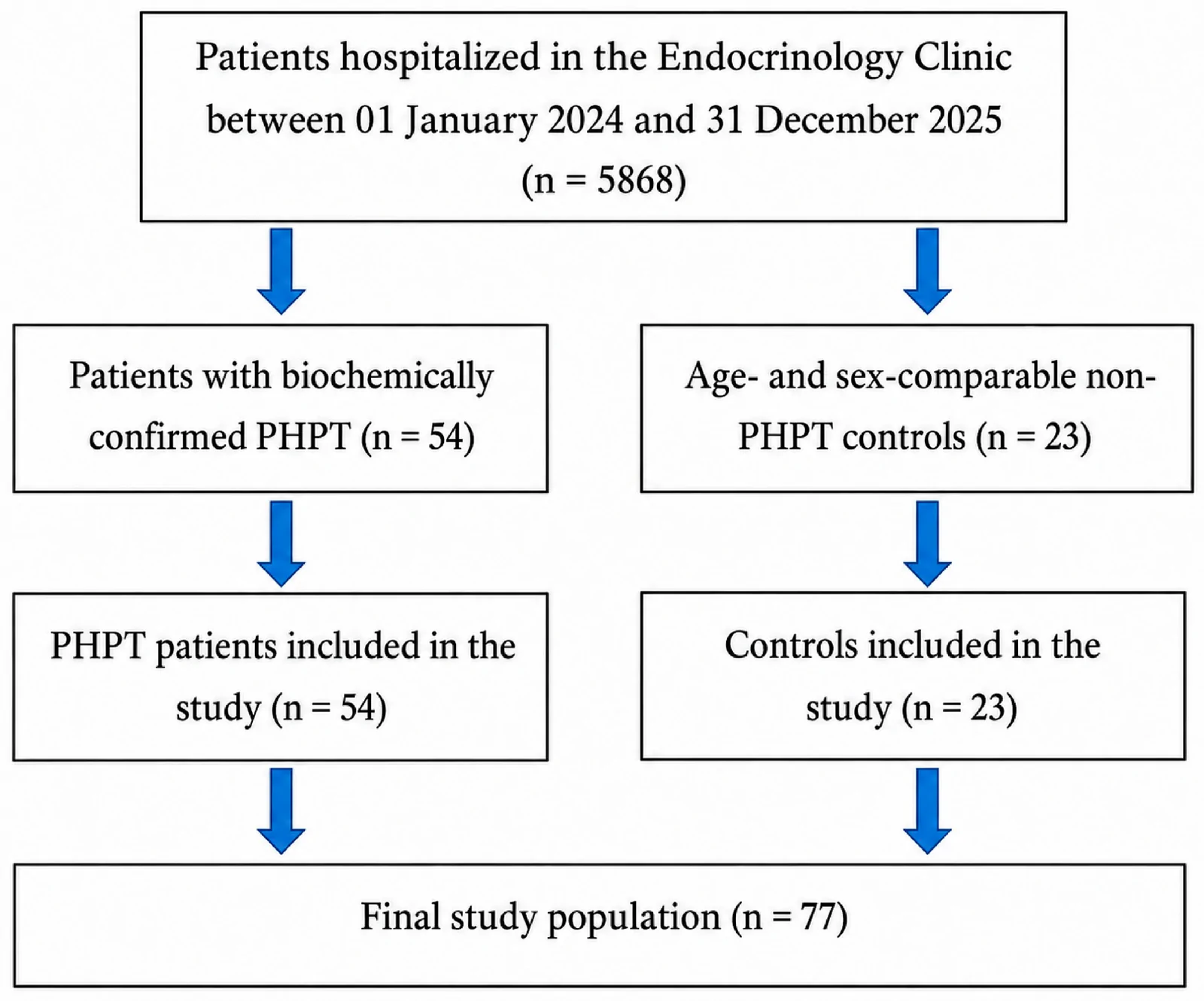
Flow diagram of participant selection and study enrollment. PHPT—primary hyperparathyroidism.

**Figure 2:**
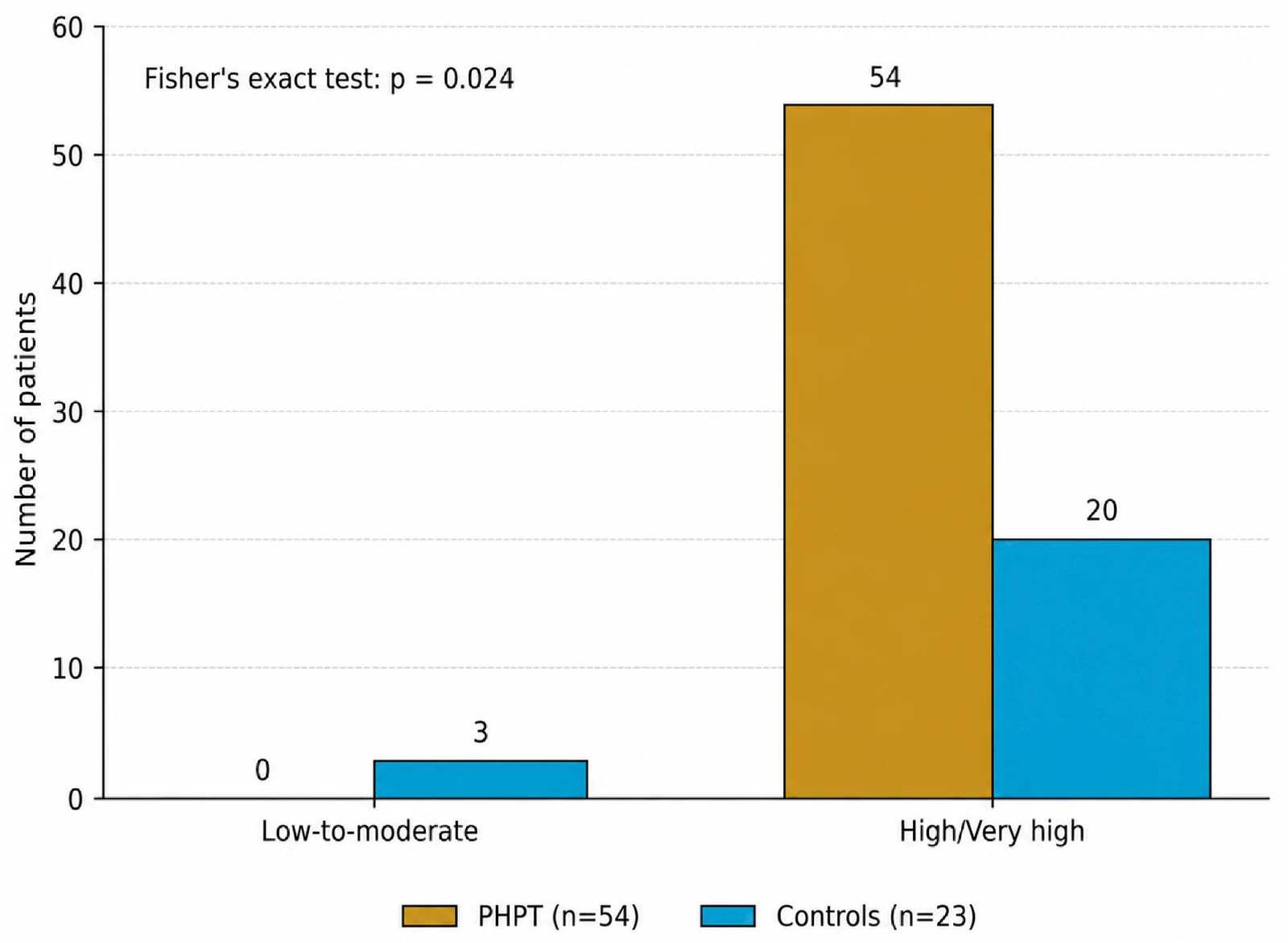
Distribution of cardiovascular risk categories according to the 2021 ESC cardiovascular risk classification. For clarity, the high- and very high-risk categories were combined. Absolute patient numbers are shown above the bars. The between-group difference was assessed using Fisher’s exact test (*p* = 0.024). PHPT—primary hyperparathyroidism.

Artificial intelligence-assisted tools (ChatGPT, OpenAI, version 5.5) were used solely to support the preparation of the graphical abstract, Figure 1 and Figure 2. The scientific content, data interpretation, and final approval of the manuscript remain the responsibility of the authors.

Figure 1 illustrates the process of participant selection and enrollment in the present study, including the identification of eligible patients with PHPT, recruitment of age- and sex-comparable controls, and inclusion in the final study population.

### 2.9. Statistical Analysis

Statistical analyses were performed using IBM SPSS Statistics software version 26.0 (IBM Corp., Armonk, NY, USA).

The distribution of continuous variables was assessed by visual inspection of histograms and using the Shapiro–Wilk test. Normally distributed variables are presented as mean ± standard deviation (SD), whereas non-normally distributed variables are expressed as median and interquartile range (IQR). Categorical variables are presented as absolute numbers and percentages.

Comparisons between patients with PHPT and controls were performed using the independent-samples Student’s *t*-test for normally distributed continuous variables and the Mann–Whitney U test for non-normally distributed variables. Categorical variables were compared using the chi-square test or Fisher’s exact test, as appropriate.

Associations between biochemical parameters related to PHPT and selected cardiovascular, ECG, and echocardiographic variables were evaluated using Pearson’s or Spearman’s correlation analysis, depending on data distribution.

Given the relatively small study population, multivariable analyses were restricted to a limited number of clinically relevant echocardiographic outcomes. Multivariable linear regression was used for selected continuous echocardiographic outcomes, whereas multivariable logistic regression was applied to selected categorical echocardiographic abnormalities. Parsimonious models were used to reduce the risk of overfitting and imprecise effect estimates. Potential confounders were selected based on their clinical relevance and observed between-group differences, with particular consideration of age, sex, BMI, HTN, and T2DM. Adjusted β coefficients or odds ratios (ORs), as appropriate, are reported together with their corresponding 95% confidence intervals (CIs).

The adjusted analyses were considered exploratory, and effect estimates were interpreted in conjunction with their 95% CIs, particularly when precision was limited by the small number of observations or outcome events. Multicollinearity among candidate covariates was assessed using variance inflation factor analysis, and variables demonstrating substantial collinearity were not included simultaneously in the same model.

All statistical tests were two-sided, and a *p*-value < 0.05 was considered statistically significant.

## 3. Results

### 3.1. Baseline Characteristics of the Study Population

The baseline demographic and clinical characteristics of the study population are presented in Table 1. The PHPT and control groups were comparable with respect to age, sex distribution, BMI, smoking status, and HTN prevalence. Patients with PHPT had a higher prevalence of T2DM and slightly higher systolic BP than controls, whereas no significant between-group differences were observed in diastolic BP, history of fractures, or other cardiovascular comorbidities.

### 3.2. Biochemical Characteristics

Table 2 presents the biochemical characteristics of the PHPT and control groups. Compared with controls, patients with PHPT exhibited the expected biochemical profile characterized by significantly higher serum PTH, total calcium, and albumin-corrected calcium concentrations, together with significantly lower serum phosphate levels.

No significant between-group differences were observed in lipid profile, renal function, thyroid function, vitamin D status, inflammatory biomarkers, or most other laboratory parameters.

**Table 2:** Biochemical characteristics of the study population.

Laboratory Parameter	PHPT (*n* = 54)	Controls (*n* = 23)	*p*-Value
PTH (pmol/L)	13.00 (9.99–16.78)	5.10 (4.12–6.37)	<0.001
Serum calcium (mmol/L)	2.70 (2.58–2.81)	2.40 (2.37–2.44)	<0.001
Ionized calcium (mmol/L)	1.39 (1.34–1.44)	1.34 (1.34–1.34)	0.386
Serum phosphate (mmol/L)	0.83 (0.75–0.95)	1.17 (1.10–1.25)	<0.001
25(OH) vitamin D (nmol/L)	67.20 (43.00–89.50)	62.00 (46.00–84.00)	0.710
Albumin (g/L)	46.00 (44.00–48.00)	46.00 (45.00–48.00)	0.452
Albumin-corrected calcium (mmol/L)	2.60 (2.50–2.70)	2.20 (2.20–2.25)	0.006
β-CTX (ng/mL)	0.56 (0.28–0.84)	1.07 (1.07–1.07)	0.381
Alkaline phosphatase (U/L)	80.00 (64.00–100.00)	57.00 (57.00–57.00)	0.267
TSH (mU/L)	2.05 (1.58–3.05)	1.98 (1.44–2.55)	0.686
Total cholesterol (mmol/L)	5.29 ± 1.11 (2.98–7.21)	5.40 ± 1.05 (3.30–7.28)	0.705
HDL cholesterol (mmol/L)	1.40 (1.27–1.71)	1.75 (1.41–1.93)	0.091
LDL cholesterol (mmol/L)	3.17 ± 1.08 (1.04–5.60)	3.27 ± 1.02 (1.14–5.61)	0.702
VLDL cholesterol (mmol/L)	0.50 (0.35–0.75)	0.40 (0.30–0.60)	0.101
Triglycerides (mmol/L)	1.16 (0.79–1.67)	0.97 (0.69–1.36)	0.128
C-reactive protein (mg/L)	1.00 (0.80–2.90)	1.10 (0.75–1.85)	0.743
NT-proBNP (pg/mL)	75.25 (29.73–141.75)	59.20 (34.85–162.50)	0.916
Serum amyloid A (µg/mL)	4.71 (3.13–7.82)	5.25 (3.67–8.90)	0.794
Endothelin-1 (pg/mL)	34.35 ± 18.17 (3.36–73.82)	29.56 ± 16.53 (3.79–62.26)	0.287
Creatinine (µmol/L)	70.67 ± 16.63 (42.00–116.00)	78.71 ± 16.11 (58.00–115.00)	0.062
eGFR (mL/min/1.73 m^2^)	85.85 ± 17.78 (43.00–119.00)	82.43 ± 18.69 (52.00–112.00)	0.475
HbA1c (%)	5.93 (5.67–6.49)	5.00 (5.00–5.00)	0.228

Values are presented as mean ± standard deviation (minimum-maximum) or median (interquartile range), as appropriate; β-CTX—beta-C-terminal telopeptide; eGFR—estimated glomerular filtration rate; HbA1c—glycosylated hemoglobin; HDL—high-density lipoprotein; LDL—low-density lipoprotein; NT-proBNP—N-terminal pro B-type natriuretic peptide; PHPT—primary hyperparathyroidism; PTH—parathyroid hormone; TSH—thyroid-stimulating hormone; VLDL—very low-density lipoprotein.

### 3.3. ECG and 24-h Holter Monitoring Characteristics

Table 3 summarizes the standard ECG and 24-h Holter monitoring findings in the PHPT and control groups. No significant between-group differences were observed in the analyzed ECG intervals and waveform parameters, including P-wave duration, PR interval, QRS duration, QTc interval, ST-segment deviation, and R-wave amplitude. During rhythm assessment, AF was observed in 8 patients with PHPT (14.8%) and in none of the controls. Supraventricular extrasystoles were recorded in 5 patients with PHPT (9.3%) and 1 control (4.3%), while conduction abnormalities were uncommon in both groups. Overall, however, the between-group difference in rhythm and conduction abnormalities did not reach statistical significance (Table 3). These findings should therefore be considered descriptive and interpreted cautiously given the small number of events (Table 3).

### 3.4. Echocardiographic Characteristics

Table 4 presents the echocardiographic characteristics of patients with PHPT and controls. Patients with PHPT showed a higher prevalence of LV hypertrophy, greater IVS and LVPW, longer transmitral flow deceleration time (DT), higher PASP, and more frequent aortic valve sclerosis/fibrotic thickening. In contrast, LV dimensions, volumes, LVEF, GLS, E/e′ ratio, RV, and the overall prevalence of diastolic dysfunction did not differ significantly between the groups.

The distribution of valvular regurgitation severity and other less frequent echocardiographic findings did not differ significantly between the PHPT and control groups (Tables S1 and S2).

### 3.5. Cardiovascular Risk

The estimated 10-year cardiovascular risk according to SCORE2 did not differ significantly between patients with PHPT and controls. The median SCORE2 value was 12.5% (IQR 7.5–27.1) in the PHPT group and 11.3% (IQR 6.1–20.4) in the control group (*p* = 0.354), indicating a comparable overall predicted cardiovascular risk despite differences in the distribution of cardiovascular risk categories.

Figure 2 summarizes the distribution of cardiovascular risk categories according to the current ESC cardiovascular risk classification [29]. For clarity, the high- and very high-risk categories were combined. All patients with PHPT (54/54) were classified as having high or very high cardiovascular risk, compared with 20/23 controls (87.0%), whereas 3/23 controls (13.0%) belonged to the low-to-moderate risk category. The between-group difference was statistically significant (*p* = 0.024).

### 3.6. Determinants of Cardiovascular Alterations in PHPT

To further explore the associations between biochemical markers of PHPT, ECG and echocardiographic parameters, and cardiovascular abnormalities in patients with PHPT, correlation and multivariable regression analyses were performed.

Correlation analysis revealed significant associations between biochemical markers and selected cardiovascular and echocardiographic parameters. The most notable findings were a positive correlation between PTH and PASP, together with inverse correlations between serum phosphate and PASP, diastolic dysfunction grade, and atrial dimensions (Table 5).

Between-group comparisons showed that patients with PHPT had greater IVS and LVPW thickness, a higher prevalence of LV hypertrophy, longer transmitral flow DT, higher PASP, and more frequent aortic valve sclerosis/fibrotic thickening than controls.

Given the relatively small study population, adjusted analyses were restricted to a limited number of clinically relevant echocardiographic outcomes. After adjustment for major potential confounders, PHPT remained associated with longer transmitral flow DT, higher PASP, aortic valve sclerosis/fibrotic thickening, and pericardial effusion (Table 6). These estimates should be interpreted cautiously in view of the limited sample size and, for categorical outcomes, the relatively small number of events.

## 4. Discussion

The present study identified several cardiovascular differences between patients with PHPT and age- and sex-comparable controls, including a higher prevalence of high or very high cardiovascular risk, T2DM, slightly higher systolic BP, more frequent LV hypertrophy and aortic valve sclerosis/fibrotic thickening, longer transmitral flow DT, and higher PASP. After adjustment for age, sex, BMI, HTN, and T2DM, PHPT remained associated with longer transmitral flow DT, higher PASP, aortic valve sclerosis/fibrotic thickening, and pericardial effusion. However, the groups differed in T2DM prevalence and systolic BP, both of which may influence cardiac structure and function. Therefore, despite multivariable adjustment, residual confounding cannot be excluded, and the findings should be interpreted as associations rather than evidence of an independent causal effect of PHPT.

These results are consistent with previous observations that PHPT is not only a disorder of calcium–phosphate and bone metabolism, but also a condition associated with cardiovascular involvement. Several reviews and observational studies have reported higher rates of HTN, LV hypertrophy, vascular stiffness, valvular and myocardial calcification, arrhythmias, and cardiovascular mortality in patients with PHPT [4,19,30]. In addition, a meta-analysis of prospective studies by Meng et al. demonstrated an association between higher circulating PTH concentrations and an increased risk of heart failure, further supporting the potential cardiovascular relevance of PTH excess [31]. Proposed mechanisms include direct effects of PTH on cardiomyocytes and vascular smooth muscle cells, as well as hypercalcemia-related alterations in vascular tone and endothelial function, disturbed intracellular calcium handling, and, with more sustained biochemical abnormalities, vascular or valvular calcification [4,19,30]. However, the European Expert Consensus on parathyroid disorders emphasizes that cardiovascular manifestations of PHPT remain incompletely defined and that their causal relationship with PHPT and their reversibility after parathyroidectomy require further prospective investigation [25]. Importantly, the clinical relevance of cardiovascular involvement in PHPT is supported by mortality data. A recent systematic review and meta-analysis by Kong et al. reported higher risks of cardiovascular death (RR 1.61, 95% CI 1.47–1.78) and all-cause death (RR 1.39, 95% CI 1.23–1.57) in patients with PHPT compared with the general population [11]. Parathyroidectomy was associated with lower cardiovascular mortality, although the observational nature and heterogeneity of much of the available evidence preclude firm causal conclusions [11].

The finding of more frequent LV hypertrophy in patients with PHPT is consistent with previous echocardiographic studies reporting increased LV mass and wall thickness in this population [30,32]. However, this finding should be interpreted cautiously, as conventional determinants of LV remodeling, including HTN, T2DM, and between-group differences in systolic blood pressure, may have contributed to the observed difference. Therefore, the present data do not allow the relative contributions of PHPT itself and coexisting cardiometabolic risk factors to LV remodeling to be clearly distinguished. This interpretation is consistent with Walker and Silverberg, who identified LV hypertrophy as one of the more consistently reported cardiovascular abnormalities in PHPT, while noting that it may be partly mediated by coexisting HTN [5].

Our diastolic findings are also clinically relevant. PHPT remained associated with longer transmitral flow deceleration time (DT), whereas the overall prevalence of diastolic dysfunction did not differ significantly between groups. The observed prolongation of DT may reflect subtle alterations in LV relaxation and filling; however, the cross-sectional design of the study does not allow determination of whether these changes are directly attributable to PHPT. Previous studies have reported inconsistent findings regarding diastolic function in PHPT, with some demonstrating impaired relaxation and others showing no clear abnormalities, particularly in patients with mild or asymptomatic disease [4,12,30]. In our cohort, preserved LVEF and only a borderline difference in GLS suggest that myocardial involvement associated with PHPT, if present, may be subtle and predominantly structural or diastolic rather than systolic. The association between higher circulating PTH concentrations and increased heart failure risk reported in the meta-analysis of prospective studies by Meng et al. further supports the potential cardiovascular relevance of PTH excess, although these data were derived from the general population rather than specifically from patients with PHPT [31]. From a clinical perspective, the grading of diastolic dysfunction reflects increasing severity of abnormalities in myocardial relaxation and LV filling pressures. Grade I is characterized predominantly by impaired relaxation and generally normal or low filling pressures, whereas Grade II represents a pseudonormal filling pattern associated with elevated LV filling pressures [27,28]. Grade III corresponds to a restrictive filling pattern, reflecting markedly reduced LV compliance and substantially elevated filling pressures, and therefore represents more advanced diastolic dysfunction [27,28]. Progression toward higher grades is clinically relevant because it reflects increasing hemodynamic impairment and may be associated with a greater likelihood of symptoms and development of heart failure with preserved ejection fraction. Nevertheless, in the present study, the absence of a significant between-group difference in the overall prevalence of diastolic dysfunction warrants cautious interpretation of these findings.

A notable finding of our study was the higher PASP observed in patients with PHPT. However, the absolute PASP values were generally within the normal or only mildly elevated range, and therefore the clinical significance of this between-group difference remains uncertain. Although the association persisted after adjustment for major potential confounders and PASP correlated positively with PTH and inversely with serum phosphate, these findings should be interpreted cautiously, particularly given the cross-sectional design. Recent experimental and clinical data suggest that PTH may be involved in pulmonary vascular remodeling and increased pulmonary pressures [33]; however, the present findings do not establish such a mechanism. In addition, pulmonary comorbidities potentially affecting PASP, including chronic bronchitis and chronic obstructive pulmonary disease, were not systematically assessed in the present study. Accordingly, the observed PASP difference should be regarded as an exploratory hemodynamic finding rather than evidence of clinically relevant pulmonary vascular disease. Larger prospective studies incorporating comprehensive pulmonary assessment are required to determine whether this association is reproducible and clinically meaningful.

Valvular involvement in PHPT has traditionally been described predominantly as calcification rather than isolated fibrosis. Earlier echocardiographic studies reported increased aortic and mitral valve calcification in patients with PHPT, particularly in those with more pronounced hypercalcemia [34]. More recently, Iwata et al. demonstrated greater aortic valve calcification even in patients with mild PHPT, with PTH emerging as a predictor of calcification burden [35]. In the present study, the recorded abnormality was increased aortic leaflet thickening and echogenicity, characterized as valve sclerosis/fibrotic thickening; valvular calcification was not separately quantified using a dedicated imaging score. Therefore, our finding should not be interpreted as evidence that isolated valvular fibrosis represents a typical or distinct manifestation of PHPT. Rather, fibrotic thickening and calcification may represent overlapping components of the broader degenerative valvular process, in which extracellular-matrix remodeling, leaflet thickening, and progressive mineralization can coexist. Accordingly, the present observation is compatible with, but not equivalent to, the well-established association between PHPT and valvular calcification.

Pericardial effusion was numerically more frequent in patients with PHPT than in controls, although the unadjusted difference was not statistically significant. An association was observed after adjustment for major measured confounders, but the limited number of events and wide confidence interval warrant cautious interpretation. A direct relationship with PHPT or altered calcium–PTH metabolism cannot be established, and alternative causes such as HF, renal or thyroid dysfunction, systemic inflammatory or infectious disorders, and malignancy should be considered. Management should therefore be guided by effusion size, symptoms, hemodynamic impact, and underlying etiology; small asymptomatic effusions generally warrant surveillance, whereas larger, symptomatic, or hemodynamically significant effusions may require more intensive evaluation and drainage. Because effusion severity was not systematically graded, this finding should be regarded as exploratory and requires confirmation in larger prospective studies.

Overall, the most consistent cardiovascular differences observed in patients with PHPT involved LV remodeling, transmitral flow DT, PASP, and aortic valve sclerosis/fibrotic thickening. These findings should be interpreted individually rather than combined into a composite measure, particularly given the limited sample size and heterogeneous nature of the abnormalities assessed. Although SCORE2 did not differ significantly between groups, standard risk algorithms may not fully capture cardiovascular abnormalities associated with PHPT.

Systematic 24-h Holter monitoring allowed assessment of rhythm abnormalities beyond a single resting ECG. AF and supraventricular extrasystoles were numerically more frequent in patients with PHPT, although the overall between-group difference was not statistically significant. Given the limited number of events, these findings remain exploratory and do not support routine rhythm surveillance; larger prospective studies using systematic ambulatory ECG monitoring are warranted.

### 4.1. Study Strengths

The main strengths of the present study include systematic identification of patients with PHPT using predefined biochemical criteria and application of the same standardized cardiovascular assessment to both groups, including clinical and biochemical evaluation, 12-lead ECG, 24-h Holter monitoring, and detailed echocardiography. The integration of biochemical markers with cardiovascular risk assessment and cardiac imaging allowed a comprehensive evaluation of the associations between PHPT-related biochemical abnormalities and cardiovascular findings.

### 4.2. Study Limitations

Several limitations should be acknowledged. The single-center cross-sectional design, relatively small and unequal study groups, and predominance of women may limit generalizability. The cross-sectional design precludes causal inference and assessment of the temporal evolution or reversibility of cardiovascular abnormalities after parathyroidectomy. The limited sample size reduced statistical power, particularly for infrequent categorical outcomes, and resulted in imprecise estimates and wide confidence intervals; therefore, multivariable analyses were restricted to selected clinically relevant outcomes and unstable secondary models were removed to reduce overfitting. Controls were selected to be comparable with the PHPT group in age and sex but not in other cardiovascular risk factors or comorbidities; differences in T2DM prevalence and systolic BP may therefore have contributed to the observed echocardiographic differences, and residual confounding cannot be excluded despite adjustment for major measured cardiovascular confounders. Pulmonary comorbidities potentially affecting PASP, including chronic bronchitis and chronic obstructive pulmonary disease, were not systematically assessed. In addition, the severity of pericardial effusion was not systematically graded using a predefined echocardiographic classification, limiting assessment of its clinical significance. These limitations should be considered when interpreting the observed associations.

### 4.3. Future Research Perspectives

Future prospective studies should further characterize the cardiovascular profile associated with PHPT using longitudinal follow-up and multimodality imaging. The pulmonary valve, which was not specifically evaluated in the present study, represents a particularly understudied area. Given the abnormalities of calcium–phosphate metabolism and PTH excess in PHPT, future studies should investigate possible pulmonary valve sclerosis, calcification, or functional abnormalities. Multimodality assessment incorporating echocardiography, cardiac magnetic resonance, and cardiac computed tomography may be particularly useful for this purpose [36].

## 5. Conclusions

In this single-center cross-sectional case–control study, patients with PHPT showed more frequent LV hypertrophy and aortic valve sclerosis/fibrotic thickening, higher PASP, and longer transmitral flow DT than age- and sex-comparable controls. After adjustment for major potential confounders, PHPT remained associated with longer transmitral flow DT, higher PASP, and aortic valve sclerosis/fibrotic thickening. These findings suggest associations between PHPT and selected structural, valvular, and hemodynamic abnormalities; however, causality cannot be inferred. Larger prospective studies are needed to confirm these associations and clarify their clinical significance and reversibility after parathyroidectomy.

## Figures and Tables

**Table 1:** Baseline demographic and clinical characteristics of the study population.

Variable	PHPT (*n* = 54)	Controls (*n* = 23)	*p*-Value
Age, years	61.0 (53.0–70.0)	60.0 (55.0–69.0)	0.969
Female sex, *n* (%)	48 (88.9%)	22 (95.7%)	0.667
BMI, kg/m^2^	27.2 (25.0–31.0)	26.5 (24.5–28.3)	0.442
HTN, *n* (%)	32 (59.3%)	11 (47.8%)	0.453
T2DM, *n* (%)	10 (18.5%)	0 (0.0%)	0.028
Heart failure, *n* (%)	0 (0.0%)	1 (4.3%)	0.299
Previous myocardial infarction, *n* (%)	0 (0.0%)	0 (0.0%)	1.000
Previous coronary revascularization, *n* (%)	0 (0.0%)	0 (0.0%)	1.000
Cerebrovascular disease, *n* (%)	3 (5.6%)	0 (0.0%)	0.550
History of fractures, *n* (%)	13 (24.1%)	2 (8.7%)	0.207
Current smoking, *n* (%)	15 (27.8%)	6 (26.1%)	1.000
Systolic BP, mmHg	132.5 (120.0–140.0)	130.0 (114.0–133.5)	0.049
Diastolic BP, mmHg	80.0 (75.8–87.5)	80.0 (74.5–80.0)	0.085

Values are presented as median (interquartile range) or number (%), as appropriate; BMI—body mass index; BP—blood pressure; HTN—hypertension; PHPT—primary hyperparathyroidism; T2DM—type 2 diabetes mellitus.

**Table 3:** ECG and 24-h Holter monitoring characteristics of patients with PHPT and controls.

Variable	PHPT (*n* = 54)	Controls (*n* = 23)	*p*-Value
P wave duration (ms)	78.2 ± 9.3 (71.9–84.5)	86.7 ± 10.4 (79.7–93.7)	0.872
PR interval (ms)	158.0 ± 5.8 (144.0–170.0)	158.8 ± 11.5 (118.0–180.0)	0.764
QRS duration (ms)	58.2 ± 6.4 (44.0–76.0)	56.2 ± 11.7 (28.0–90.0)	0.486
QTc interval (ms)	406.6 ± 29.5 (341.0–503.0)	419.4 ± 26.8 (370.0–470.0)	0.100
ST segment deviation (mV)	0.012 ± 0.057 (−0.130–0.160)	0.004 ± 0.048 (−0.070–0.160)	0.565
R wave amplitude (mV)	1.18 ± 0.60 (0.13–3.06)	1.18 ± 0.70 (0.16–2.89)	1.000
Rhythm and conduction disorders, *n* (%)			0.122
AF	8 (14.8%)	0	
Supraventricular extrasystoles	5 (9.3%)	1 (4.3%)	
Left bundle branch block	1 (1.9%)	0	
Right bundle branch block	0	1 (4.3%)	

Values are presented as mean ± standard deviation (minimum-maximum) or number (%), as appropriate; AF—atrial fibrillation; PHPT—primary hyperparathyroidism.

**Table 4:** Echocardiographic characteristics of the study population.

Echocardiographic Parameter	PHPT	Controls	*p*-Value
Aortic root diameter, PLAX, mm	30.0 (29.0–33.0)	31.0 (29.5–34.5)	0.453
LA diameter, PLAX, mm	35.0 (32.2–38.0)	34.0 (31.0–37.5)	0.421
LA longitudinal diameter, apical 4-chamber, mm	49.5 (45.2–53.0)	48.0 (46.0–49.5)	0.328
RA longitudinal diameter, apical 4-chamber, mm	47.0 (44.0–52.0)	46.0 (43.0–49.0)	0.435
LV end-systolic diameter, PLAX, mm	28.0 (24.0–30.0)	28.0 (24.0–29.0)	0.557
LV end-diastolic diameter, PLAX, mm	44.0 (38.2–47.0)	44.0 (40.5–47.0)	0.836
LV end-systolic volume, mL	34.0 (25.0–42.0)	29.0 (23.0–36.5)	0.225
LV end-diastolic volume, mL	92.5 (79.0–106.8)	90.0 (77.5–98.5)	0.336
LVEF, %	64.0 (59.0–68.0)	67.0 (61.5–70.0)	0.110
LV GLS, %	−17.4 (−19.6 to −15.3)	−19.6 (−20.9 to −16.7)	0.066
IVS thickness, mm	10.0 (9.0–11.0)	9.0 (8.0–10.5)	0.024
LVPW thickness, mm	10.0 (9.0–11.0)	9.0 (8.0–10.0)	0.015
LV hypertrophy, present	31 (57.4%)	7 (30.4%)	0.046
Mitral E-wave, m/s	0.7 (0.6–0.8)	0.7 (0.6–0.8)	0.530
Mitral A-wave, m/s	0.7 (0.6–0.9)	0.6 (0.5–0.8)	0.088
Mitral flow DT, ms	281.5 (245.8–332.5)	222.0 (191.0–244.0)	<0.001
Mitral e′, m/s	0.08 (0.07–0.12)	0.11 (0.07–0.13)	0.572
Mitral a′, m/s	0.10 (0.09–0.13)	0.11 (0.09–0.13)	0.578
E/e′ ratio	7.3 (5.6–9.2)	6.3 (4.8–8.8)	0.403
Mitral s′, m/s	0.09 (0.08–0.11)	0.09 (0.07–0.11)	0.787
RV end-diastolic diameter, PLAX, mm	28.0 (26.2–29.8)	28.0 (25.0–29.5)	0.902
PASP, mmHg	24.5 (21.0–29.0)	19.0 (17.5–21.5)	<0.001
Diastolic dysfunction, any grade	21 (38.9%)	8 (34.8%)	0.801
Grade I	20 (37.0%)	6 (26.1%)	
Grade II	1 (1.9%)	1 (4.3%)	
Grade III	0 (0.0%)	1 (4.3%)	
Mitral valve sclerosis/fibrotic thickening	19 (35.2%)	5 (21.7%)	0.292
Aortic valve sclerosis/fibrotic thickening	21 (38.9%)	3 (13.0%)	0.032
Pericardial effusion	21 (38.9%)	4 (17.4%)	0.109

Values are presented as median (interquartile range) or number (%), as appropriate; DT—deceleration time; IVS—interventricular septum; GLS—global longitudinal strain; LA—left atrial; LV—left ventricular, LVEF—left ventricular ejection fraction; LVPW—left ventricular posterior wall; PASP—pulmonary artery systolic pressure; PLAX—parasternal long axis view; RA—right atrial; RV—right ventricular.

**Table 5:** Correlations between biochemical markers of primary hyperparathyroidism and cardiovascular parameters.

Biochemical Parameter	Associated Variable	*n*	Spearman ρ	*p*-Value
Serum phosphate	PASP	61	−0.475	<0.001
PTH	PASP	60	0.352	0.006
Serum calcium	LVPW thickness	59	0.292	0.025
Serum phosphate	Diastolic dysfunction grade	74	−0.255	0.029
Serum calcium	Systolic BP	74	0.237	0.042
Serum phosphate	LA diameter	62	−0.257	0.043
Albumin-corrected calcium	Diastolic dysfunction grade	52	0.277	0.047
Serum phosphate	RA apical diameter	60	−0.257	0.047

Spearman’s rank correlation analysis was used to evaluate associations between biochemical markers of PHPT and selected cardiovascular or echocardiographic parameters; only statistically significant correlations are shown; BP—blood pressure; LA—left atrial; LVPW—left ventricular posterior wall; PASP—pulmonary artery systolic pressure; PHPT—primary hyperparathyroidism; PTH—parathyroid hormone; RA—right atrial.

**Table 6:** Multivariable associations between PHPT and selected echocardiographic outcomes.

Echocardiographic Outcome	Adjusted Estimate for PHPT	95% CI	*p*-Value
Transmitral flow DT	β = +45.0 ms	13.3 to 76.7	0.005
PASP	β = +4.26 mmHg	1.51 to 7.02	0.002
Aortic valve sclerosis/fibrotic thickening	OR = 5.90	1.39 to 25.08	0.016
Pericardial effusion	OR = 4.22	1.17 to 15.19	0.028

Adjusted β coefficients were derived from multivariable linear regression models for continuous outcomes, whereas adjusted odds ratios were derived from multivariable logistic regression models for categorical outcomes. All models were adjusted for age, sex, BMI, HTN, and T2DM. CI—confidence interval; DT—deceleration time; HTN—hypertension; PASP—pulmonary artery systolic pressure; PHPT—primary hyperparathyroidism; T2DM—type 2 diabetes mellitus.

## Data Availability

The data supporting the results of this research are available from Stefan Naydenov (snaydenov@gmail.com) upon reasonable request, subject to applicable ethical and privacy restrictions.

## Supplementary Materials

The following supporting information can be downloaded at: https://www.mdpi.com/article/10.3390/jcm15176605/s1, Table S1: Comparison of echocardiographic valvular findings in patients with primary hyperparathyroidism and controls. Table S2: Comparison of other echocardiographic findings between patients with PHPT and controls.

## Abbreviations

The following abbreviations are used in this manuscript:

AF	Atrial fibrillation
ASCVD	Atherosclerotic cardiovascular disease
BP	Blood pressure
β-CTX	Beta-C-terminal telopeptide
BMI	Body mass index
CKD	Chronic kidney disease
CVD	Cardiovascular disease
ECG	Electrocardiogram
ESC	European Society of Cardiology
eGFR	Estimated glomerular filtration rate
GLS	Global longitudinal strain
HbA1c	Glycosylated hemoglobin
HDL	High-density lipoprotein
HF	Heart failure
HTN	Hypertension
LA	Left atrial
LDL	Low-density lipoprotein
LV	Left ventricular
LVEF	Left ventricular ejection fraction
LVPW	Left ventricular posterior wall
NT-proBNP	N-terminal pro B-type natriuretic peptide
PASP	Pulmonary artery systolic pressure
PHPT	Primary hyperparathyroidism
PTH	Parathyroid hormone
RA	Right atrial
RV	Right ventricular
T2DM	Type 2 diabetes mellitus
TSH	Thyroid-stimulating hormone
VLDL	Very low-density lipoprotein
