# Supplementary material for: Cardiovascular Alterations in Primary Hyperparathyroidism: Associations with Risk Profile, Cardiac Structure, and Function
Source: J Clin Med. 2026 Aug 26;15(17):6605. doi: 10.3390/jcm15176605 (PMC13566182; doi:10.3390/jcm15176605)
Supplement: Supplementary file 1 [file jcm-15-06605-s001.zip › Table S1.pdf]

Table S1. Comparison of echocardiographic valvular findings in patients with primary hyperparathyroidism and controls.

Variable	PHPT (*n* = 54)	Controls (*n* = 23)	*p*-value
Mitral regurgitation, *n* (%)			
Mild	37 (68.5%)	13 (56.5%)	
Moderate	0 (0.0%)	0 (0.0%)	0.180
Severe	1 (1.9%)	0 (0.0%)	
Aortic regurgitation, *n* (%)			
Mild	12 (22.2%)	5 (21.7%)	
Moderate	1 (1.9%)	0 (0.0%)	0.846
Severe	0 (0.0%)	0 (0.0%)	
Tricuspid regurgitation, *n* (%)			
Mild	42 (77.8%)	16 (69.6%)	
Moderate	0 (0.0%)	1 (4.3%)	0.904
Severe	1 (1.9%)	0 (0.0%)	
