# Supplementary material for: Cardiovascular Alterations in Primary Hyperparathyroidism: Associations with Risk Profile, Cardiac Structure, and Function
Source: J Clin Med. 2026 Aug 26;15(17):6605. doi: 10.3390/jcm15176605 (PMC13566182; doi:10.3390/jcm15176605)
Supplement: Supplementary file 1 [file jcm-15-06605-s001.zip › Table S2.pdf]

Table S2. Comparison of other echocardiographic findings between patients with PHPT and controls.

Other echocardiographic findings	PHPT (n = 54)	Controls (n = 23)	p-value
MV prolapse	16 (29.6%)	4 (17.4%)	0.395
LA, RA and RV dilatation	3 (5.6%)	4 (17.4%)	0.187
Atrial septal aneurysm	4 (7.4%)	1 (4.3%)	1.000
PFO	2 (3.7%)	0 (0.0%)	1.000
LA and RA dilatation	2 (3.7%)	0 (0.0%)	1.000
RV dilatation	1 (1.9%)	0 (0.0%)	1.000
LA, RA, RV and Ao root dilatation	1 (1.9%)	0 (0.0%)	1.000
Aneurysm of the ascending aorta	1 (1.9%)	0 (0.0%)	1.000
LA dilatation	0 (0.0%)	1 (4.3%)	0.299

Ao – aortic; LA – left atrial; MV – mitral valve; PFO – patent foramen ovale; RA – right atrial, RV – right ventricle.
